# Bioactive Secondary Metabolites from Fungi of the Genus *Cytospora* Ehrenb. (Ascomycota)

**DOI:** 10.3390/molecules28073120

**Published:** 2023-03-31

**Authors:** Boris Yotta Kianfé, Joseph Tchamgoue, Abolfazl Narmani, Rémy Bertrand Teponno, André-Ledoux Njouonkou, Marc Stadler, Simeon Fogue Kouam

**Affiliations:** 1 Department of Chemistry, Faculty of Science, University of Dschang, Dschang P.O. Box 67, Cameroon; 2 Department of Organic Chemistry, Faculty of Science, University of Yaounde I, Yaounde P.O. Box 812, Cameroon; 3 Department of Chemistry, Higher Teacher Training College, University of Yaounde I, Yaounde P.O. Box 47, Cameroon; 4 Department of Plant Protection, Faculty of Agriculture, University of Tabriz, Tabriz 51666, Iran; 5 Department of Plant Sciences, Faculty of Science, University of Bamenda, Bambili P.O. Box 39, Cameroon; 6 Department of Microbial Drugs, Helmholtz Centre for Infection Research and German Centre for Infection Research (DZIF), Partner Site Hannover/Braunschweig, Inhoffenstrasse 7, 38124 Braunschweig, Germany

**Keywords:** *Valsa*, canker, natural products, Cytosporaceae, bioactive compounds

## Abstract

*Cytospora* is a genus of fungi belonging to the Cytosporaceae family (Sordariomycetes, Ascomycota) considered as a prolific source of specialized metabolites due to their ability to produce diverse secondary metabolites with a broad range of biological activities. Since the first chemical investigation of this genus in the 1980s, further studies have led to the isolation and structural elucidation of several bioactive compounds including cytosporones, nonanolides, macrocyclic dilactones, and terpenoids. This review summarizes, for the first time, the chemical diversity of bioactive secondary metabolites from the genus *Cytospora* and highlights its potential as an alternative source of secondary metabolites for pharmacological studies. Moreover, this review will serve as a basis for future investigations of compounds of this genus.

## 1. Introduction

Significant progress has been made in the control of many diseases caused by infectious microorganisms. Recurrent epidemics due to drug-resistant microorganisms and the appearance of new microbial pathogenic strains mandate the discovery of new antibiotics [1]. Fungi are growing tremendously in terms of their repertoire of biosynthetic and pharmaceutical chemistry as they greatly enrich structurally novel secondary metabolites with promising bioactivities. In their natural environment, fungi must compete for resources and it has been hypothesized that this competition likely induces the biosynthesis of secondary metabolites for defence. Only about 7% of fungi have been studied for the chemistry of secondary metabolites [2]. Among the studied fungi species, those of the genus *Cytospora* are also reputed to be a prolific source for the generation of structurally versatile and biologically significant metabolites. They are particularly known for the production of potent antimicrobial compounds [3,4].

The genus *Cytospora*, which is currently classified in the class Sordariomycetes, order Diaporthales, and family Cytosporaceae, was first introduced by Ehrenberg in 1818 with four species (*C. betulina*, *C. epimyces*, *C. resinae*, and *C. ribis*) [5]. *Cytospora* is one of the most important groups of plant pathogenic fungi on over 130 species of monocotyledonous, dicotyledonous, and gymnosperm hosts (e.g., Anacardiaceae, Elaeagnaceae, Fabaceae, Juglandaceae, Myrtaceae, Rosaceae, Salicaceae, and Ulmaceae) in the world [6,7].

To date, approximately 688 species epithets of *Cytospora* have been listed in Index Fungorum [8]. However, most of these species names were considered synonyms of previously described taxa and lack herbarium materials, ex-type cultures, and DNA sequence data. The validity of many of these needs to be carefully tested. Currently, several sexual genera such as *Leucocytospora*, *Leucostoma*, *Valsella*, and *Valseutypella* were considered as synonyms of *Valsa* [8]. Based on the “One Fungus = One Name” taxonomic framework, *Cytospora* (the older asexual typified name) was proposed as the recommended name over *Valsa* (the younger sexual typified name) and Cytosporaceae took priority over Valsaceae [9,10].

*Cytospora* species are mainly associated with dieback and stem canker (known as *Cytospora*-, *Leucostoma*-, *Valsa*-, or perennial canker) in over 130 species of woody hosts [9,11]. *Cytospora chrysosperma* (Teleomorph: *Valsa sordida*), a type of species of *Cytospora*, is regarded as the most important and virulent causal agent of canker disease on various plant hosts distributed worldwide, including *Populus*, *Fraxinus*, *Malus*, *Prunus*, *Ulmus*, etc. [9,12,13].

A total of about 150 species of *Cytospora* have been discovered and described on canker of woody plants [14]; among these, species such as *C. ambiens*, *C. ceratosperma*, *C. cincta*, *C. chrysosperma*, *C. eucalypticola*, *C. leucosperma*, *C. leucostoma*, *C. mali*, and *C. germanica* have been reported from various hosts repeatedly [13,15,16,17]. In spite of the fact that *Cytospora* species are mainly considered weak pathogens, they negatively affect the productivity and longevity of fruit trees worldwide and lead to chronic wood infections in various hosts such as *Populus* spp. [16], *Malus* spp. [13], *Vitis vinifera* [15], *Olea europaea* [18], and *Quercus* sp. [17]. It has been demonstrated that *Cytospora* species are highly destructive and virulent on *Prunus* and *Populus* species [15,16].

**Figure 1:**
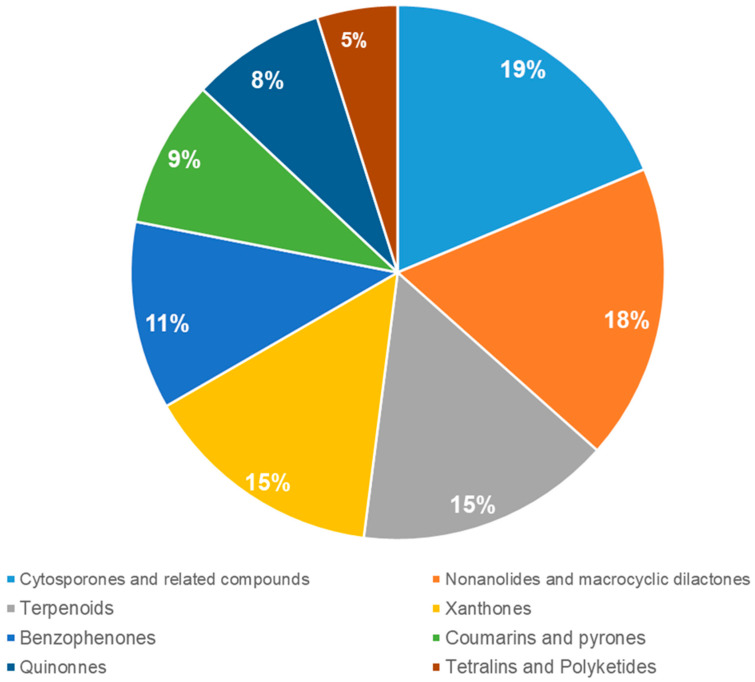
The distribution of isolated compounds of the genus *Cytospora*.

They have diverse ecological habits, including saprotrophs that colonize dead or decaying woods, seemingly innocuous endophytic strains isolated from the xylem, bark, and leaves of symptomless woody plants [19,20], and parasites with destructive canker pathogens causing dieback and stem canker of woody plants [9,13]. The biological and ecological diversity of this genus has made it a potent source of bioactive secondary metabolites. Extensive work carried out on its chemical and pharmacological potentials has led to the isolation and structural elucidation of many bioactive compounds (Figure 1) including xanthones, quinones, and coumarins [4,7,21,22]; benzophenones [23,24,25,26]; macrolides [27]; and terpenoids [28,29]. Possibly, these compounds have some role in plant–pathogen interactions and play a role as pathogenicity factors in pathogenesis. Since these compounds are not host-specific toxins (HSTs), they cannot be considered the main determinants of pathogenicity but contribute to virulence of the producing pathogen [30]. However, to the best of our knowledge, there is no comprehensive review summarizing the bioactive compounds isolated from this genus. This review thus provides up-to-date information on the study of biologically active secondary metabolites produced by various species of the genus *Cytospora* and its synonyms over the last four decades, from 1980 to 2021.

## 2. Xanthones, Quinones, and Coumarins

**Figure 2:**
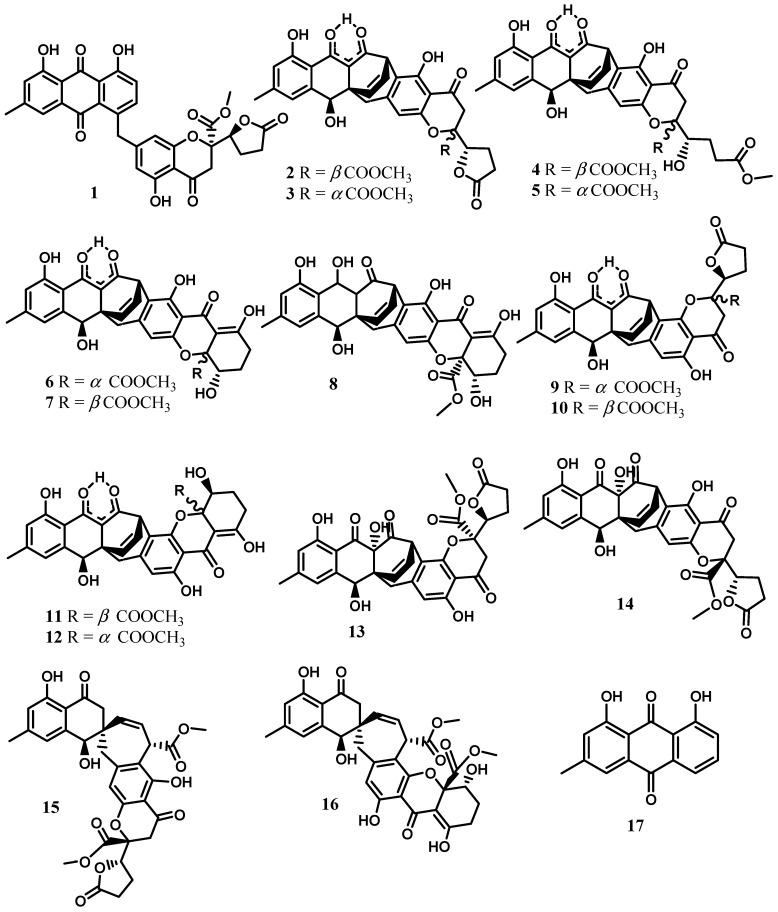

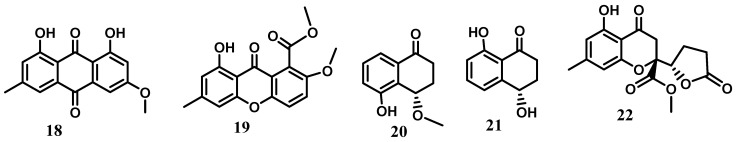
Xanthones, quinones, and coumarins from the genus *Cytospora*.

The chemical investigation of extracts from cultures of *Cytospora eugeniae* BCC42696 obtained from the petiole of *Arenga pinnata* Merr. led to the isolation and structural elucidation of twenty-two compounds including cytosporanthraxanthone (**1**); xanthoquinodins A7–A10 (**3**, **5**, **7**, and **14**); ketoxanthoquinodin A6 (**8**); xanthoquinodins B6-B8 (**9**, **10**, and **13**); spiroxanthoquinodins A (**15**) and B (**16**); and one synthetically known derivative, 2-methoxy pinselin (**19**). The known compounds were identified as xanthoquinodins A4-A6 (**2**, **4**, and **6**), B4 (**11**), and B5 (**12**); chrysophanol (**17**); physcion (**18**); (4*S*)-5-hydroxy-4-methoxy-α-tetralone (**20**); (4*S*)-4,8-dihydroxy-α-tetralone (or isosclerone) (**21**); and gonytolide C (**22**) [4]. Xanthoquinodins A6 (**6**), B4 (**11**), and B5 (**12**) exhibited strong activity against *Plasmodium falciparum* (IC_50_ 0.52–0.92 mM) and displayed significant activity against *Bacillus cereus* (MIC 1.56 mg/mL). In addition, xanthoquinodins A4 (**2**), A6 (**6**), B4 (**11**), and B5 (**12**) and ketoxanthoquinodin A6 (**8**) showed cytotoxicity against both cancerous (MCF-7, KB, and NCI-H187) and noncancerous (Vero) cells [4]. In fact, xanthoquinodins A4-A6 (**2**, **4**, **6**), B4 (**11**), and B5 (**12**) were originally isolated from the endolichenic fungus *Chaetomium elatum* [31]. They were reported to show cytotoxicity against human myeloid leukemia (HL-60), hepatocellular carcinoma (SMMC-7721), lung cancer (A-549), breast cancer (MCF-7), and colon cancer (SW480) cells with IC_50_ values in a range from 2.04 to >40 mM. The anthraquinone derivatives chrysophanol (**17**) and physcion (**18**) are commonly found in various medicinal plants of the genera *Rheum* [32,33] and *Rumex* [34,35,36] belonging to the family Polygonaceae. Both compounds were reported to possess a wide range of biological activities including antifungal [37], hepatoprotective [32], anti-inflammatory [36,38,39], and antioxidant [40], as well as cytotoxic, activities against various cancerous cells [36,38]. (4*S*)-5-hydroxy-4-methoxy-α-tetralone (**20**) previously isolated from the fruits of *Juglans mandshurica* [41] showed weak cytotoxicity against human liver cancer cells (HepG-2) with an IC_50_ value of 88.23 μM [42]. Concerning isosclerone (**21**), it was obtained for the first time from the fungus *Sclerotinia sclerotiorum* [43] and was further isolated from *Phaeoacremonium aleophilum* and *P*. *chlamydosporum* [44] involved in grapevine disease just as the *Cytospora* species. Gonytolide C (**22**) was originally isolated from the fungus *Gonytrichum* sp. and was inactive at 10 mg/mL for innate immune response activity using the ex vivo *Drosophila* culture system [22] (Figure 2).

**Figure 3:**
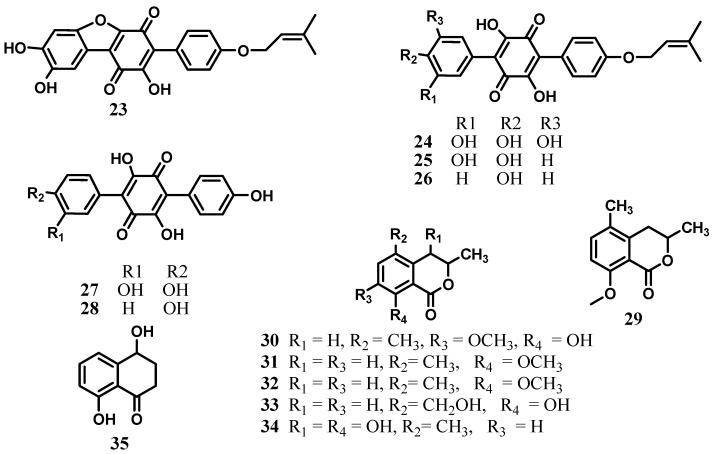
Terphenylquinones and dihydroisocoumarin derivatives from the genus *Cytospora*.

From the extracts of the walnut tree pathogenic fungus *Cytospora* sp. CCTU A309, the terphenylquinone derivatives, cytosporaquinones A–D (**23**–**26**), leucomelone (**27**), and atromentin (**28**) were isolated [22]. Cytosporaquinones A–D (**23**–**26**) exhibited significant cytotoxicity against the mouse fibroblast L929 and cervix carcinoma KB-3-1 cell lines, with IC_50_ values ranging from 2.4 to 26 µg/mL. Furthermore, cytosporaquinones B–D (**24**–**26**), and leucomelone (**27**) showed interesting antiviral activity against the hepatitis C virus (HCV), while only moderate antimicrobial effects were observed against twelve bacterial strains, with IC_50_ values ranging from 16.66 to 66.66 µg/mL [22]. Leucomelone (**27**) and atromentin (**28**) previously obtained from the solid state fermentation of an unidentified fungus F010248 isolated from a soil sample collected in a corn field around Kongju city (Korea) were shown to be the first inhibitors specific to enoyl-ACP reductase (FabK) of *Streptococcus pneumoniae* [45]. The dihydroisocoumarin derivative 3,5-dimethyl-8-methoxy-3,4-dihydroisocoumarin (**29**) was also obtained from *Cytospora* sp. strain CCTU A309 [22], while its congeners 3,5-dimethyl-8-hydroxy-7-methoxy-3,4-dihydroisocoumarin (**30**), 3,5-dimethyl-8-methoxy-3,4-dihydroisocoumarin (**31**), 3,5-dimethyl-8-hydroxy-3,4-dihydroisocoumarin (5-methylmellein, **32**), 8-hydroxy-5-hydroxymethyl-3-methyl-3,4-dihydroisocoumarin (5-hydroxymethylmellein, **33**), 4,8-dihydroxy-3,5-dimethyl-3,4-dihydroisocoumarin (4-hydroxy-5-methylmellein, **34**), and tetralone 4,8-dihydroxy-1-tetralone (isosclerone, **35**) were isolated from a culture filtrate of *Cytospora eucalypticola* [46] (Figure 3). The fungus was originally isolated from the bark of *Eucalyptus perriniana* cv. Spinning Gum (Myrtaceae) growing at the Royal Botanic Gardens, Kew [46]. These isocoumarins are mildly antifungal and antibacterial towards Gram-positive bacteria. Nevertheless, in the antifeedant assay against the larvae of *Spodoptera littoralis*, 5-hydroxymethylmellein (**33**) showed activity at 100 ppm (feeding index (mean S.E.M.) 42.1 ± 6.42; *p* < 0.05), whereas 5-methylmellein (**32**) did not influence any feeding (feeding index, −30.1 ± 20.14; *p* > 0.05), suggesting that C-5 substituent influences the behavioural response of the larvae [46].

The naphthalenone derivative (**36**) and the dihydroisocoumarin congener (3*R*)-5-methylmellein (**37**) are among the secondary metabolites obtained from *Cytospora* sp. isolated from the plant *Ilex canariensis* [47]. (3*R*)-5-methylmellein (**37**) was discovered for the first time in *Fusicoccum amygdale* as the main phytotoxic metabolite [48] and later widely reported from endophytic fungi, pathogenic fungi, marine-derived mangrove fungi, and the heartwood of the Fijian species *Euphorbia fidjiana* [47,49]. The chemical investigation of the fermentation broth of the filamentous fungus *Cytospora* sp. (MF 6608) isolated from leaf litter of *Manilkara bidentata* collected from Puerto Rico led to the discovery of cytosporic acid (**38**). which inhibited the strand transfer reaction of HIV-1 integrase with an IC_50_ of 20 µM [3]. Cytosporacin (**39**), a novel antibacterial polyketide containing naphthopyranone and isochromandione moieties, was isolated from the fermentation broth of the fungus *Cytospora rhizophorae* obtained from the roots of two species of the mangrove tree *Rhizophora mangle* and *R. racemosae* growing in Florida by He and collaborators [50]. This compound showed moderate activity in vitro against Gram-positive bacteria. The minimum inhibitory concentrations obtained by the broth dilution method were 32 µg/mL for *Staphylococcus aureus* (two strains, including a methicillin-resistant strain), 16 µg/mL for *Enterococcus faecium*, and 32 µg/mL for *Bacillus subtilis* [50]. A biosynthetic ^13^C-labeling experiment indicated that cytosporacin was derived from acetate origin. Two bisanthraquinones, namely cytoskyrins A (**40**) and B (**41**), were isolated from fermentation broths of the fungus *Cytospora* sp. CR200 which was obtained from a branch of *Conocarpus erecta* in the Guanacaste National Park, Costa Rica [21]. Cytoskyrin A exhibited potent in vitro antibacterial against Gram-positive bacteria with MICs ranging from 0.03 to 0.25 μg/mL and DNA-damaging activities (10 ng/spot), whereas cytoskyrin B was inactive in these assays. Some compounds related to cytoskyrins A and B include (+)-epicytoskyrin produced by the endophytic fungus *Diaporthe* sp. isolated from a tea plant [51], as well as luteoskyrin and rugulosin obtained from *Penicillium islandicum* and *Penicillium rugulosum* [52].

**Figure 4:**
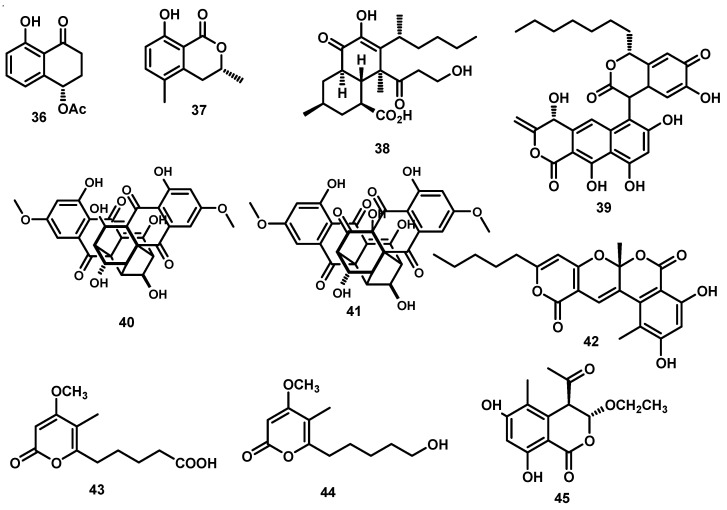
Dihydroisocoumarin, pyranone, bisanthraquinone, pyrone, and isocoumarin derivatives from the genus *Cytospora*.

The pyrone and isocoumarin hetero-dimers that possess an unprecedented skeleton with a polyoxygen-hetero 6/6/6/6 tetracyclic fused ring system named cytospone A (**42**) and three of its biosynthetic precursors, cytospones B–D (**43**–**45**), were isolated from *Cytospora rhizophorae* A761 [7] (Figure 4). The structure of cytospone A represents the first family of natural hetero-dimers comprising pyrone and isocoumarin moieties [7].

## 3. Benzophenones

**Scheme 1:**
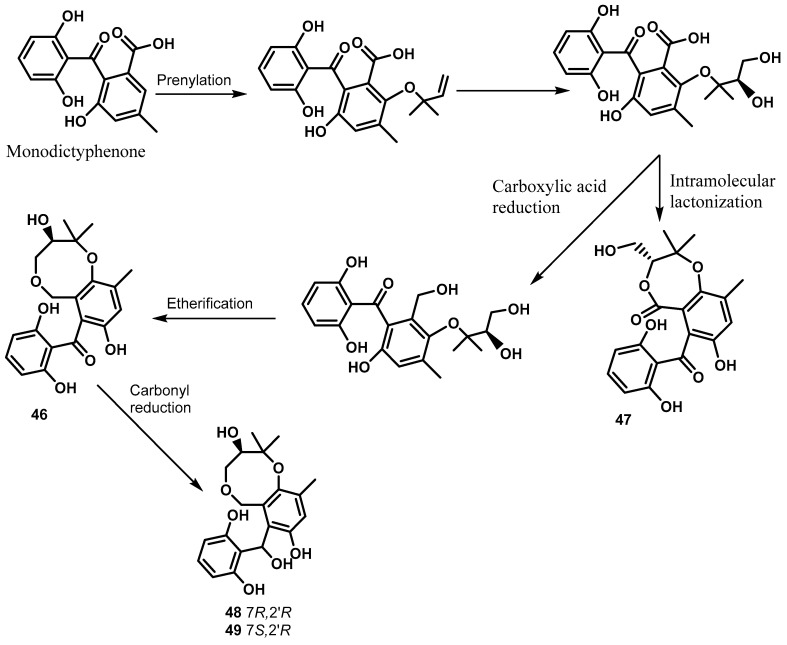
Proposed biogenetic pathway of cytosporins A–D [23].

Cytosporins A–D (**46**–**49**) are hemiterpene-conjugated phenolics with an unprecedented benzo[b][1,5]dioxocane skeleton produced by the fungus *Cytospora rhizophorae* A761 derived from *Morinda officinalis* How (Rubiaceae) [23]. They represent the first examples of natural meroterpenoids which bear a benzo[b][1,5]dioxocane framework embodying hemiterpene and benzophenone moieties [23]. As discussed by Liu and collaborators [23], cytosporins A–D are generated through the elaboration of a formally unified precursor that might be envisaged from the well-known fungal metabolite monodictyphenone by the installation of the densely functionalized hemiterpene nucleus through chemoselective prenylation and stereoselective dihydroxylation. They also demonstrated the intramolecular lactonization of the precursor that would directly transform it into cytosporin B, whereas cytosporin A might be accessible from the carboxylic acid reduction followed by etherification. Moreover, cytosporins C and D could be readily deduced to be generated from cytosporin A by a simple carbonyl reduction (Scheme 1) [23]. These compounds were shown inactive against the bacteria *Escherichia coli* and *Staphylococcus aureus* even at a concentration of 250 μg/mL [23].

**Scheme 2:**
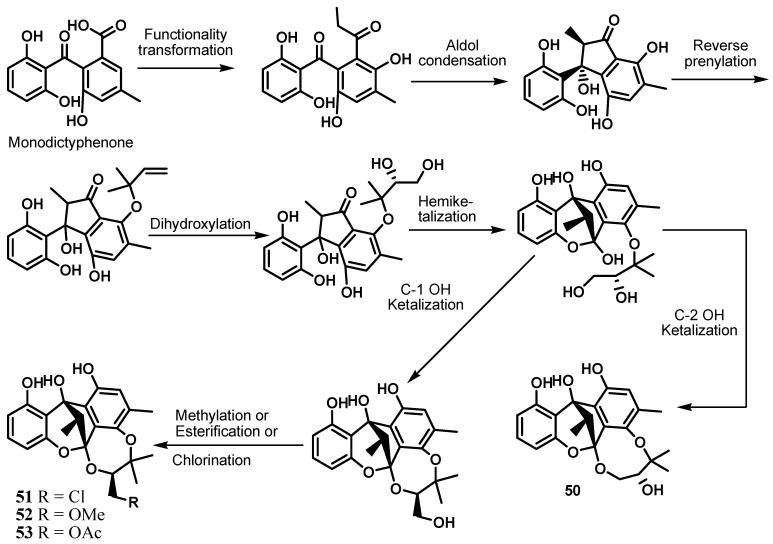
Proposed biogenesis of cytorhizins A−D [24].

Further investigation of *Cytospora rhizophorae* A761 led to the discovery of four novel polyketide heterodimers sharing an unprecedented 6/6/5/6/8 or 6/6/5/6/7 pentacyclic ring system and fused as a fascinating cage-like skeleton, embodying a polyoxygenated isopentyl unit and a highly structure-combined benzophenone scaffold trivially named cytorhizins A–D (**50**–**53**) [24]. Cytorhizins B and D showed weak cytotoxic activity against HepG-2, MCF-7, SF-268, and A549 cell lines with IC_50_ values ranging from 29.4 to 68.6 μM [24]. However, cytorhizins A–D were found to be devoid of any significant antimicrobial activity even at a concentration of 100 μM [24]. Inspired by their structural characteristics, a hypothetical biogenetic pathway embracing intriguing aldol condensation and dihydroxylation/selective ketalization cascade reaction as key biosynthetic steps was proposed as shown in Scheme 2 [24].

Two axially chiral benzophenones featuring an epoxy isopentyl unit and a propionyl moiety, rhizophols A (**54**) and B (**55**), were among the metabolites produced by *Cytospora rhizophorae* A761 [25]. Rhizophol B was disclosed as a pair of inseparable atropisomers (4:3, ^1^H NMR integration) in acetone-d_6_. Moreover, temperature-dependent NMR experiments, crystal X-ray diffraction, and quantum molecular mechanical calculation were conducted to illustrate the existence of the rapidly interconverting diastereoisomers and the absolute structure [25]. Rhizophol A was proved to be a promising lead compound for novel antioxidant drug development with an IC_50_ of 13.07 ± 0.94 μM and no cytotoxicity at 100 μM (DPPH radical scavenging) [25].

**Figure 5:**
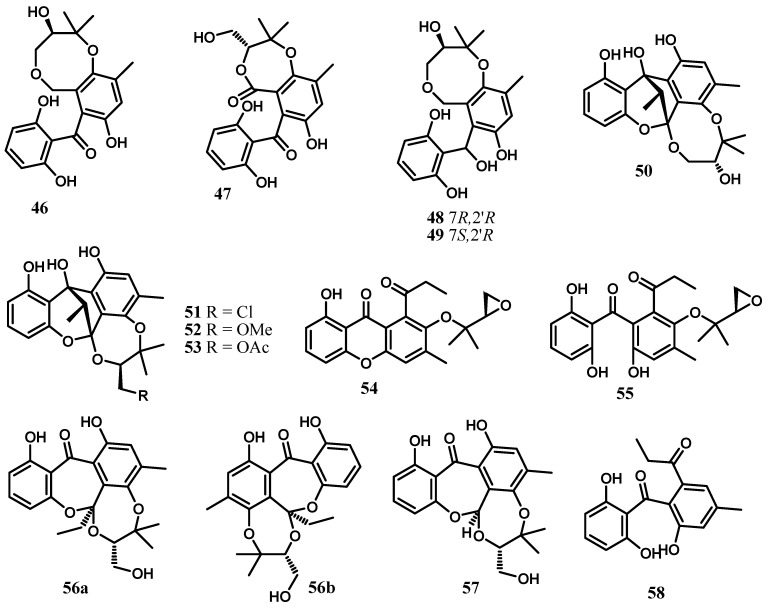
Benzophenones from the genus *Cytospora*.

A pair of novel enantiomeric benzophenone-hemiterpene adducts, (+)-cytorhizophin A (**56a**) and (−)-cytorhizophin A (**56b**), together with cytorhizophin B (**57**) and their biosynthetically related precursor cytorhizophin C (**58**), were obtained from *Cytospora rhizophorae* A761 [26] (Figure 5). These compounds were evaluated for their antimicrobial activities against the bacteria *Escherichia coli* and *Staphylococcus aureus* and were found to be devoid of significant activity even at a concentration of 100 µg/mL [26].

## 4. Nonanolides and Macrocyclic Dilactones

Naturally occurring nonanolides are a large class of secondary metabolites with an interesting 10-membered macrolide subunit. Metabolites of the nonanolide family can be roughly divided into two groups according to the structural features of the side chain: simple nonanolides with a methyl group at C-9 and nonanolides with extended alkyl chains at C-9. Structurally complex nonanolides with additional rings are sometimes also included in the family [27].

Cytospolides A–E (**59**–**63**) possessing an unprecedented 15-carbon skeleton with the unique chemical feature of a C-2 methyl group were isolated from *Cytospora* sp., obtained from Ilex canariensis [27]. Their structures were elucidated by spectroscopic analysis, chemical interconversion, and single-crystal XRD. The structure of the solution and solid state conformers were compared by experimental methods (X-ray, NMR), as well as by solvent and gas phase DFT calculations [27]. The results of the cytotoxicity activity revealed that the 8-O-monoacetate and the absolute configuration at C-2 clearly play important roles in the growth inhibition of the tumour line. Further chemical investigation of this fungus led to the discovery of fourteen nonanolide derivatives named cytospolides F–Q (**64**–**75**) and decytospolides A and B (**76**, **77**) [53]. Furan containing nonanolides **71**, **72**, **73**, and **75** showed considerably cytotoxic activity against A-549 cells [53]. The free hydroxyl groups at C-3 and C-4 seem to play an important role in the cytotoxic activity since the inhibition remarkably decreased in **73**, lacking the two secondary hydroxyl groups. The activity of decytospolide A (**76**) increased dramatically when the 8-OH was acetylated [53].

**Figure 6:**
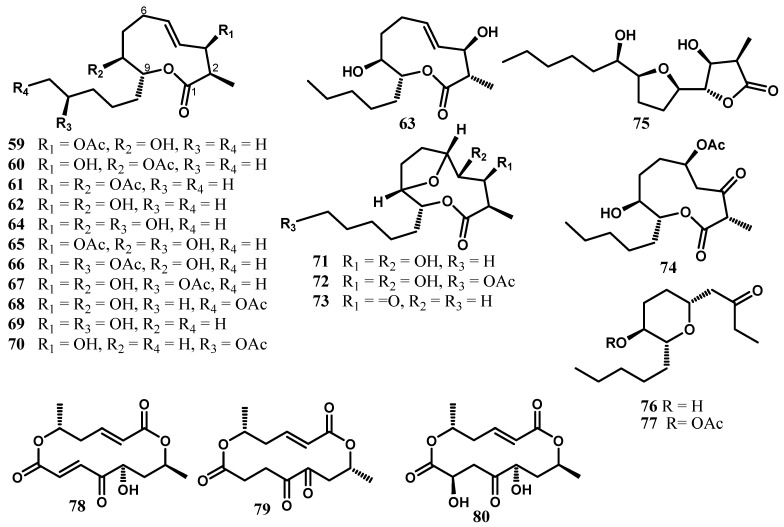
Nonanolides and macrocyclic dilactones from the genus *Cytospora*.

Grahamimycins A (**78**), Al (**79**), and B (**80**) are three macrocyclic dilactones obtained in crystalline form from *Cytospora* sp. Ehrenb isolated from *Pinus contorta* var. *Latifolia* Egelm (Figure 6). Grahamimycin A exhibited activity against thirty-six species of bacteria, eight species of blue-green algae, and two species of green algae, as well as antifungal activity against five fungi [54]. Grahamimycin A1 was also among the macrodiolides isolated from the marine fungus *Varicosporina ramulosa* [55].

## 5. Cytosporones and Related Compounds

**Scheme 3:**
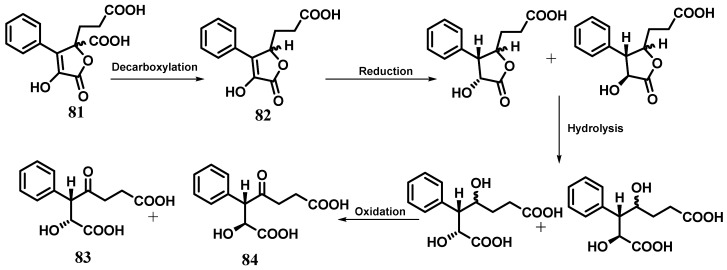
Proposed biogenetic pathway for the formation of compounds **83** and **84** from butenolides **81** and **82**.

The butenolides 3-(2-carboxy-2,5-dihydro-4-hydroxy-5-oxo-3-phenyl-2-furyl)propionic acid (**81**) and 3-(2,5-dihydro-4-hydroxy-5-oxo-3- phenyl-2-furyl)propionic acid (**82**) and the dioic acids (2*R*,3*R*) 2-hydroxy-3-phenyl-4-oxoheptanedioic acid (**83**) and (2*S*,3*S*) 2-hydroxy-3-phenyl-4-oxoheptanedioic acid (**84**) are among the secondary metabolites produced by the fungus *Cytospora* sp. CCTUA309. Compounds **81** and **82** were tested for their nematicidal activity, but no significant mortality was observed [22]. These metabolites were previously produced by the marine-derived fungus *Talaromyces rugulosus* isolated from the Mediterranean sponge *Axinella cannabina* [56]. Due to the structural similarity between the two heptanedioic acid derivatives **83** and **84** and butanolides **81** and **82**, a biogenetic pathway (Scheme 3) for the formation of heptanedioic acid derivatives from butanolides implying decarboxylation, reduction, hydrolysis, and selective oxidation was proposed [22].

Cytosporones A–E (**85**–**89**) are octaketides obtained from the fermentation broth of the fungus *Cytospora* sp. CR200 [21]. Compounds D (**88**) and E (**89**) exhibited activity against Gram-positive bacteria with MICs values of 4–8 μg/mL, but they were inactive in the biochemical induction assay [21].

The EtOAc fraction from *Cytospora* sp. TT-10 obtained from twigs of the Japanese oak was subjected to chemical study, leading to the isolation of a new cytosporone analog (**90**) together with integracin A (**91**) and cytosporone N (**92**) [57]. These compounds were evaluated for their antimicrobial activities using the paper disk method but only cytosporone N and integracin A showed a moderate antimicrobial activity against *Raffaelea quercivora* JCM 11526 with MIC values of 12 and 13 μg/mL, respectively [57]. Integracin A obtained from *Cytospora* sp. isolated from *Ilex canariensis* was reported to show HIV-1 inhibitory activity [58]. This fungus also produced integracin B (**93**) and other antimicrobial derivatives, namely (*R*)-5-((*S*)-hydroxy(phenyl)-methyl)dihydrofuran-2(3H)-one (**94)** and its 6-acetate (**95**), together with (*S*)-5-((*S*)-hydroxy(phenyl)-methyl)dihydrofuran-2(3H)-one (**96**), (*S*)-5-benzyl-dihydrofuran-2(3H)-one (**97**), 5-phenyl-4-oxopentanoic acid (**98**), γ-oxo-benzenepentanoic acid methyl ester (**99**), and 3-(2,5-dihydro-4-hydroxy-5-oxo-3-phenyl-2-furyl)propionic acid (**82**). These compounds showed a moderate antimicrobial activity against the fungi *Microbotryum violaceum*, *Botrytis cinerea*, and *Septoria tritici*; the alga *Chlorella fusca*; and the bacterium *Bacillus megaterium* [47].

**Figure 7:**
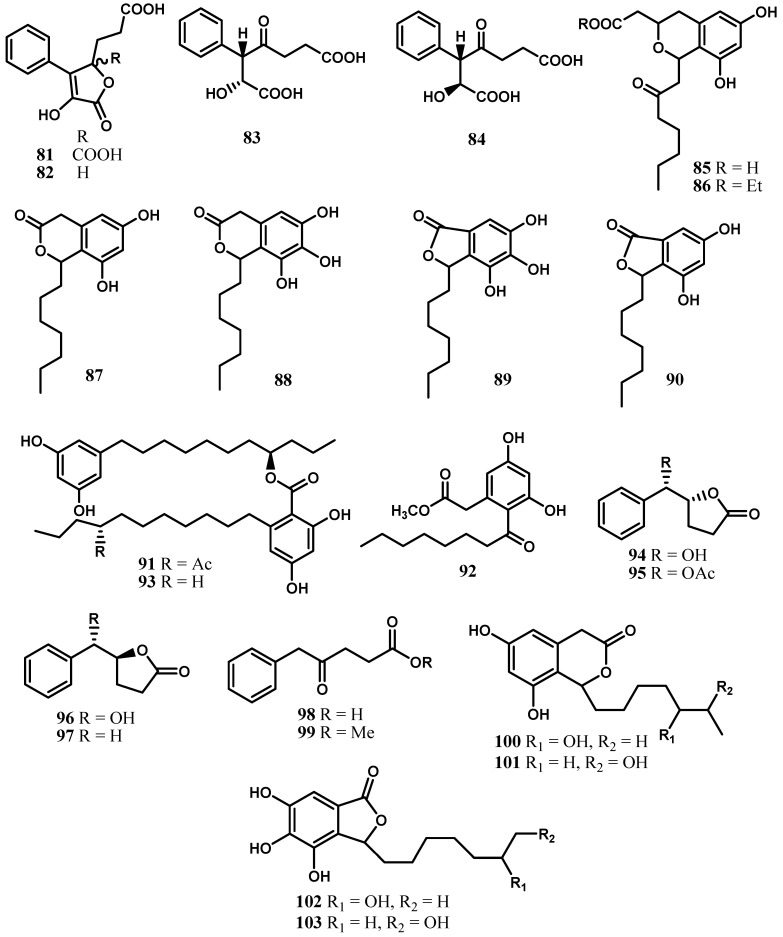
Cytosporones and related compounds from the genus *Cytospora*.

Cytosporones O (**100**), P (**102**), and Q (**103**), as well as dothiorelone H (**101**), were obtained from the ascomycete fungus *Cytospora* sp. CML 1841 (=IBT 41593) isolated from *Phoradendron perrottetii* collected in Brazil [59] (Figure 7).

## 6. Terpenoids

Terpenoids represent a large and diverse bioactive class of secondary metabolites produced by various plants and fungi. Accumulating evidence on their broad spectrum of biological activities coupled with a tolerable toxicity profile has sparked renewed interest with regard to their application, especially in cancer treatment [60]. Several compounds belonging to this class were reported from *Cytospora* species.

The chemical investigation of *Cytospora* sp. isolated from Chinese mangrove *Ceriops tagal* yielded a new bicyclic sesquiterpene seiricardine D (**104**) and known metabolites, namely xylariterpenoids A (**105**) and B (**106**), regiolone (**35**) (22*E*, 24*R*)5,8-epidioxy-5α,8α-ergosta-6,22*E*-dien-3-*β*-ol (**107**), (22*E*, 24*R*)5,8-epidioxy-5α,8α-ergosta-6,9(11),22-trien-3-*β*-ol (**108**), *β*-sitosterol (**109**), and stigmast-4-en-3-one (**110**) [29]. The antimicrobial activities of compounds **112**–**110** against four human pathogens—*Escherichia coli*, methicillin-resistant *Staphylococcus aureus* (MRSA), *Pseudomonas aeruginosa*, and *Candida albicans*—and three plant pathogens—*Bacillus subtilis*, *Colletotrichum gloeosporioides,* and *Magnaporthe* (= *Pyricularia*) *oryzae*—were reported. Compounds **104**–**110** exhibited a weak inhibitory activity against *Magnaporthe oryzae*, with MICs of 58.3–839.6 µM [29]. Xylariterpenoids A (**105**) and B (**106**) were previously isolated from the deep-sea-derived fungus *Graphostroma* sp. MCCC 3A00421 [61]. Ergosterol and its derivatives are ubiquitous in fungal cell membranes, where they regulate permeability and fluidity. (22*E*,24*R*)5,8-epidioxy-5α,8α-ergosta-6,22*E*-dien-3-*β*-ol (**107**) and stigmast-4-en-3-one (**110**) were previously obtained from the Chinese mangrove *Rhizophora mucronata* endophytic fungi, *Pestalotiopsis* sp. [42], while (22*E*,24*R*)5,8-epidioxy-5α,8α-ergosta-6,9(11),22-trien-3-*β*-ol (**108**) was also isolated from the gorgonian *Eunicella cavolini* and the ascidian *Trididemnum inarmatum* [62].

**Figure 8:**
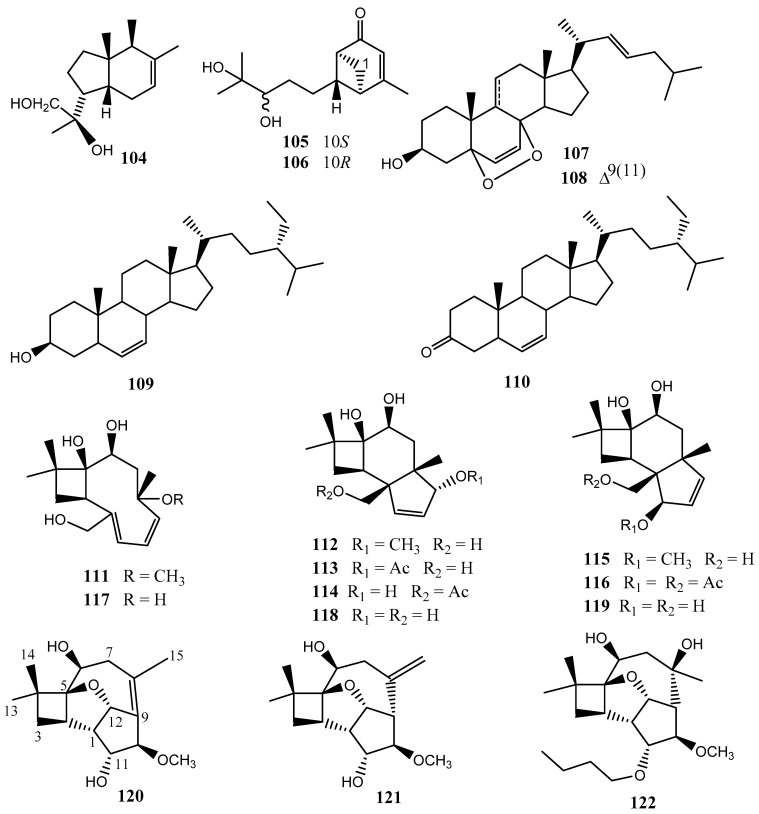
Terpenoids from the genus *Cytospora*.

Nine caryophyllene sesquiterpenoids named punctaporonins N–S (**111**–**116**) and 6-hydroxypunctaporonins B (**117**), A (**118**), and E (**119**) have been isolated from the solid cultures of *Cytospora* sp. obtained from a soil sample collected at Linzhi, Tibet, China. Compounds **112**, **115**, and **116** exhibited moderate cytotoxicity against HeLa (cervical epithelium) cells with IC_50_ values of 16.6, 10.4, and 47.4 µM, respectively [63]. Five other caryophyllene sesquiterpenoids named cytosporinols A–C (**120**–**122**) were previously isolated from solid cultures of the same fungal strain by Li and collaborators [28] (Figure 8). The structures of cytosporinols A–C were elucidated by NMR spectroscopy, and the absolute configurations of the C-11 secondary alcohol in cytosporinol A and the 6,8-diol moiety in cytosporinol C were deduced using the modified Mosher and Snatzke’s method [28]. Furthermore, the configuration of cytosporinol C was confirmed by a single crystal X-ray crystallographic analysis. These compounds were evaluated for their cytotoxic activities. Cytosporinols B (**121**) and C (**122**) showed moderate cytotoxicity against HeLa (cervical epithelium) cells, with IC_50_ values of 16.5 and 21.1 µM, respectively, while the positive control cisplatin showed an IC_50_ value of 7.6 µM [28].

Although the compounds described in this review possessed very interesting biological properties, they have been isolated in very small amounts. However, the development of drugs requires large quantities of lead compounds, which can be obtained by fermentation. Nevertheless, some secondary metabolites isolated from fungi of the genus *Cytospora* have already been synthesized by organic chemists, as summarized in Table 1 below.

## 7. Conclusions and Recommendations

The present review indicates 122 compounds consisting mainly of xanthones, quinones, coumarins, benzophenones, macrolides, and terpenoids isolated from fungi of the genus *Cytospora*. These secondary metabolites were evaluated in most cases for their antimicrobial and cytotoxic activities, and several of these compounds were found to be very effective. Hence, these fungi represent an important source of bioactive compounds that could be used as leads in the development of antibiotics and anticancer drugs. Surprisingly, most of the studied *Cytospora* species were not fully identified. Taking into consideration their diversity and richness in the production of bioactive secondary metabolites, it is recommended that mycologists pay more attention to the taxonomy of species of this genus, which will certainly lead to the discovery of several new strains and to the efficient exploration of their biotechnological potentials. Moreover, a more diligent search on the occurrence of the reported compounds in other plant pathogenic fungi should be conducted as this may give a more realistic picture on the ecological function of these compounds. Additional studies should also be carried out on this genus using omics and co-culturing strategies to shed more light on the bioactive compounds species that this genus could produce.

## Figures and Tables

**Table 1:** Synthesis of some secondary metabolites produced by fungi of the genus *Cytospora*.

Compounds	Reactions	References
Chrysophanol (**17**) Physcion (**18**)	Synthesis from 1-amino-5-chloroanthraquinone and 2-methylanthraquinone	[64]
Isosclerone (**21**)	Biotransformation of the commercially available 5-hydroxy-1,4-naphthalenedione using endophytic fungus *Paraconiothyrium variabile*	[65]
Gonytolide C (**22**)	Stereoselective total synthesis by aldol reaction between acetophenone and butyrolactone containing α-keto ester, followed by diastereoselective intramolecular cyclization	[66]
Leucomelone (**27**)	Synthesis from 2,5-dibromo-3,6-dimethoxy-1,4-benzoquinone	[67]
Atromentin (**28**)	Total synthesis based on double Suzuki–Miyaura coupling	[68]
Cytoskyrin A (**40**)	Total synthesis of 2,2′-*epi*-cytoskyrin A	[69]
Cytospolides D (**62**)	Total synthesis from *L*-malic acid and *D*-mannitol	[70]
Grahamimycin A (**78**)	Total synthesis via Yamaguchi macrocyclization	[71]
Grahamimycin A1 (**79**)	Total synthesis from 5(*R*)-hydroxy-2(*E*)-hexenoate and 7(*R*)-octynoic acid	[72]
Cytosporone B (**86**)	Total synthesis from 1-bromo-3,5-dimethoxybenzene	[73]
Cytosporone E (**89**)	Synthesis via the Meyers ortho-alkylation of a chiral aromatic oxazoline	[74]

## Data Availability

Not applicable.
